# Mentalizing under stress and psychotic experiences: An experience sampling study

**DOI:** 10.1111/papt.70048

**Published:** 2026-02-27

**Authors:** Ercan Ozdemir, Angus MacBeth, Helen Griffiths

**Affiliations:** ^1^ Section of Clinical Psychology, School of Health in Social Science University of Edinburgh Edinburgh UK

**Keywords:** affective symptoms, alexithymia, emotion regulation, mentalization, psychosis

## Abstract

**Aim:**

Theoretical models identify stress‐induced transient disruptions in the capacity to mentalize as a risk marker for psychosis yet there is little research that takes into account the context dependence of mentalizing and psychotic processes. To increase ecological validity, we investigated how mentalizing and psychotic experiences were concurrently and longitudinally associated in the flow of daily life, hypothesizing that mentalizing difficulties would predict both concurrent and subsequent presentations of psychotic experiences.

**Method:**

An analogue sample responded to self‐report assessments of situational stress, momentary mentalizing difficulties in understanding one's feelings, negative affect and psychotic experiences, using a 1‐week experience sampling schedule with eight measurement points per day. Concurrent and lagged associations between mentalization, negative affect and psychotic experiences were estimated via linear mixed effects and vector autoregressive modelling.

**Results:**

The analysed sample (*n* = 43) identified as 63% female, 21% male and 16% non‐binary with all participants aged between 18 and 38 years. Thirty per cent of the sample self‐reported a personal history of psychosis, and 37% were receiving mental health support. Significant concurrent and cross‐lagged associations of positive effect sizes were identified between mentalizing difficulties and psychotic experience severity.

**Conclusion:**

Mentalizing capacity may decline under stress and accordingly influence the severity and persistence of psychotic experiences. Our small sample size and the gender distribution may limit the generalizability of the findings. Future research should integrate interview‐ or performance‐based metrics of mentalizing ability into longitudinal designs enabling more extensive examination of different domains of mentalizing difficulties and psychotic experiences.

## INTRODUCTION

Psychotic experiences can be defined transdiagnostically with the psychometric structure remaining invariant across subclinical to clinical presentations (Pedrero & Debbané, 2017; Van Os & Reininghaus, 2016). Usually structured along the dimensions of negative (constrictions in affective range and interpersonal engagement) and positive symptoms (thought disorder, delusions and hallucinations), psychotic experiences are relatively common in the general population, with the highest incidence and prevalence rates observed in adolescence (Staines et al., 2022). The persistence of psychotic experiences into young adulthood increases risk for developing psychotic disorders (Healy et al., 2019; Staines et al., 2023; Van Os & Reininghaus, 2016). Understanding subclinical but persistent psychotic experiences could offer a probabilistic pathway to psychosis, enhancing understanding of risk/resilience factors, transition to psychosis and opportunities for early prevention (e.g., McGorry et al., 2024).

Persistence of psychotic experiences can be understood through the latent trait framework of schizotypy, wherein loosening of associations (i.e., cognitive slippage) functions as a central indicator of underlying vulnerability (Meehl, 1962, 1990). Conversely, from a network perspective, persistence can be conceptualized as emerging from (i) patterns of co‐activation among the structural components of psychotic experiences and (ii) how strongly these components reinforce each other as a response to stimuli (e.g., stress, thought disorder, delusions and social withdrawal co‐occurring and sustaining each other) (Borsboom, 2017; Van Os & Reininghaus, 2016). Often viewed in opposition, schizotypy and network models share important commonalities, with both assuming a similar structure for psychotic experiences and incorporating a positive domain that encompasses perceptual aberrations, magical ideation, paranoia and unusual self‐experiences (Mark & Toulopoulou, 2016; Mason, 2015). Importantly, both models identify traumagenic pathways marked by affective dysregulation and interpersonal aversion, generating vulnerability to psychosis transdiagnostically, independent of traditional schizophrenia‐specific predispositions (Meehl, 1962, 1990; Van Os & Reininghaus, 2016).

Psychotic symptom formation due to heightened negative affective reactivity to stress has been conceptualized as the affective pathway to psychosis (Myin‐Germeys et al., 2003; Myin‐Germeys & van Os, 2007). This paradigm identifies affective dysregulation (i.e., amplified emotional and physiological reactivity to everyday stressors) as a mediator of the relationship between childhood trauma and psychosis (Alameda et al., 2020; Klippel et al., 2017; Radhakrishnan et al., 2019). This heightened reactivity increases vulnerability to acute elevations in psychotic experiences particularly under conditions of social or emotional adversity. Thus, affective dysregulation may serve as a proximal mechanism through which distal developmental insults translate into psychosis risk. However, systematic review evidence indicates a small but inconsistent positive association between affective dysregulation and psychotic experiences as they unfold in daily life; this underscores the need to delineate the underlying mechanisms that sustain or modulate these associations across contexts and time (Bortolon & Nardelli, 2025; Muddle et al., 2021).

Mentalization, the ability to understand mental states of self and others, has been postulated as an affect regulatory capacity influenced by childhood adversity and is implicated in the development of psychosis (Debbané et al., 2016; Liotti & Gumley, 2008). Disruptions in mentalizing processes can be characterized by stress‐reactive arrests in reflective functioning, presenting as inabilities to identify a discrete emotion and/or synthesize discrete experiences into organized narratives of experience (Luyten et al., 2020; Lysaker et al., 2019). Mentalizing difficulties show pervasive associations with psychotic experiences involving thought disorder (Lehmann & Ettinger, 2023; Myers et al., 2024) and negative symptoms (McGuire et al., 2023), and strengthen the associations between positive symptoms and psychological distress (Faith et al., 2023). Difficulties in identifying feelings may also link persistent subclinical positive psychotic experiences to negative arousal (Ozdemir et al., 2025a), highlighting mentalizing capacity as a factor underpinning the associations between distress and psychotic experiences.

Difficulties in identifying feelings can be viewed through the lens of alexithymia, which denotes chronic disruptions in mentalizing capacity as marked by a profile of reflective disengagement from emotional experiences and constricted imagination (Taylor et al., 2024). Alexithymia is commonly conceptualized as a relatively stable trait involving persistent difficulties in identifying and describing feelings and externally oriented thinking (Taylor & Bagby, 2013). Relative stability is a between‐person metric of change in alexithymic traits under situational demands, indicating that individuals retain their rank‐order position over time, consistent with conceptualizing alexithymia as a trait‐like vulnerability rather than a transient state. However, such transient states of reflective dysfunction due to stress and arousal have been implicated in acute psychotic crisis, marked by the collapse of an already vulnerable reflective capacity (Liotti & Gumley, 2008). Therefore, state sensitivity of mentalizing processes may hold significant clinical value for psycho‐social interventions.

**FIGURE 1:**
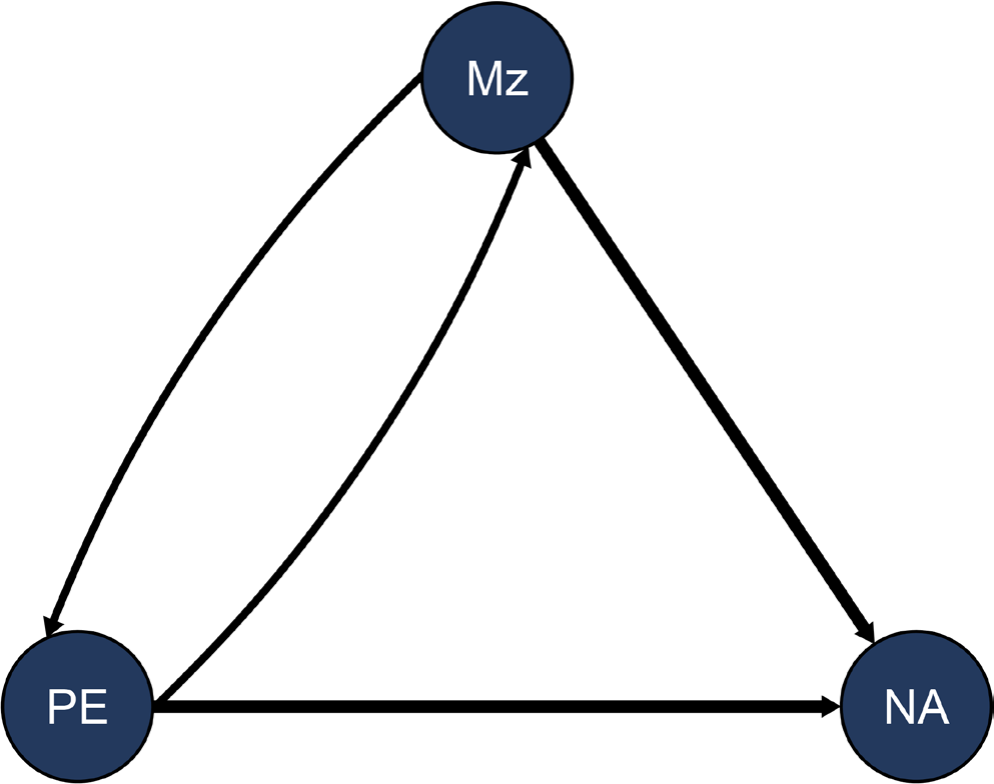
Network representation of the theoretical model. Mz, mentalizing; NA, negative affect; PE, psychotic experiences.

From a methodological perspective, evidence for the role of mentalizing difficulties in the *development* of psychotic experiences has been limited by a reliance on cross‐sectional designs (e.g., Ozdemir, Xiao, et al., 2025). Better estimation of the impact of disruptions in mentalization capacity requires ecologically valid measurement methods that capture experiences as they unfold moment‐to‐moment in daily life, such as experience sampling methods (ESM; Hermans et al., 2019). One ESM study reports alexithymia as a moderator of the relationship between affect regulation and psychotic experiences (Kimhy et al., 2020). A more recent ESM study identifies stress as a predictor of concurrent and subsequent mentalizing difficulties (Höller et al., 2025), yet moment‐to‐moment associations between mentalizing difficulties and psychotic experiences are still to be investigated. Bringing these strands together, the current ESM study aimed to examine the concurrent and cross‐lagged associations between mentalization, negative affect and psychotic experiences. Figure 1 is a network representation of the theoretical model postulating negative affect as an outcome of the synergistic interactions between mentalizing difficulties and psychotic experiences. From these premises, we hypothesized that mentalizing difficulties will predict the severity of concurrent and subsequent psychotic experiences, controlling for the persistence of psychotic experiences and concurrent negative affect.

## METHODS

### Participants

The sample was recruited from the participant pool of a longitudinal cohort study (Ozdemir et al., 2024) and through online advertisements posted on psychosis support groups. The sample for the longitudinal cohort study was recruited through online advertisements aimed at reaching individuals from the general population experiencing psychological distress. Eligibility was limited to residents of English‐speaking countries such as Australia, Canada, Ireland, New Zealand, South Africa, the United Kingdom and the United States. A total of 1110 individuals completed the baseline assessment between September and December 2021, who were invited to participate in the current study as part of cohort follow‐up. Additional details regarding recruitment procedures and participant characteristics are available in Ozdemir et al. (2024) and Ozdemir et al. (2025b).

Inclusion criteria were English fluency, being aged 18 years and above and ownership of a smartphone. Individuals experiencing a mental health crisis requiring in‐patient treatment were not eligible to take part. Participants providing fewer than 17 valid ESM assessments were excluded post hoc to secure sufficient within‐person variability for stable estimation of temporal effects in the analyses.

### Design

An experience sampling method (ESM) was used with the ethical approval obtained from the University of Edinburgh Health in Social Science ethics committee. The ESM procedure involved a baseline survey and a 19‐item momentary assessment survey created in *Qualtrics* and embedded in the notification tool, *Samply* (Shevchenko et al., 2021). Participants who provided informed consent completed a baseline survey. Momentary assessment began the day after enrolment, consisting of 56 notifications across 1 week. Each day between 10:00 and 22:00, participants received eight notifications, pseudo‐randomly spaced at least 40 min apart. Each ESM survey link remained active for 15 min following the notification.

### Measures

#### Baseline assessment

##### Demographics

The demographic questionnaire collected information on age, gender, employment status and included three questions assessing: (i) whether the participant or a close family member had ever experienced a psychotic episode, (ii) whether the participant had a personal history of psychosis, and (iii) whether they were currently undergoing mental health treatment. These were used to characterize mental health history in the sample.

##### Negative symptoms

The Self‐Evaluation of Negative Symptoms (SENS; Dollfus et al., 2016) is a 20‐item self‐report tool for assessing negative symptoms, with items rated on a 3‐point scale. The SENS demonstrates acceptable convergent validity through its associations with interview‐based negative symptom scales, as well as divergent validity, indicated by its lack of associations with measures of insight, parkinsonism and positive symptoms (Dollfus et al., 2022). The reliability index of the SENS was excellent in the current study (α = .92).

##### Disorganized schizotypy

The disorganized schizotypy subscale of the Multidimensional Schizotypy Scale Brief (MSS‐B; Gross, Kwapil, Burgin, et al., 2018; Gross, Kwapil, Raulin, et al., 2018) consists of 12 items answered in a yes/no format. This subscale has demonstrated acceptable convergent validity, shown through its association with the disorganized schizotypy subscale of the Schizotypal Personality Questionnaire (Gross, Kwapil, Burgin, et al., 2018). Reliability of the disorganization subscale of the MSS‐B was excellent in the current study (α = .92).

##### Paranoia

The Paranoia checklist (Freeman et al., 2005), an 18‐item self‐report instrument with a 5‐point rating scale (‘1 = Do not believe it’ to ‘5 = Absolutely believe it’), was used to evaluate the conviction in which paranoid beliefs are held and its convergent validity was supported by the association with the Paranoia Scale. The reliability index of the Paranoia Checklist was excellent (α = .95).

##### Mentalization

The Reflective Functioning Questionnaire‐8 (RFQ; Fonagy et al., 2016) and the Toronto Alexithymia Scale (TAS; Bagby et al., 1994) were used to examine mentalizing difficulties. The RFQ is an eight‐item self‐report measure rated on a seven‐point scale ranging from 1 (strongly disagree) to 7 (strongly agree). Associations between the mental state uncertainty subscale and assessments of self‐harm, psychotic experiences and affective dysregulation suggest acceptable construct validity (Fonagy et al., 2016). However, the RFQ's reliability was questionable in the current study (α = .61). The TAS, on the other hand, evaluates mentalizing difficulties in identifying and describing feelings and externally oriented thinking. Research supports a three‐dimensional structure for the TAS (Schroeders et al., 2022), while the externally oriented thinking subscale yields questionable reliability metrics (Kooiman et al., 2002). The TAS as a composite measure showed acceptable reliability in the current study (α = .70).

#### The ESM survey

Table 1 presents the 19‐item ESM survey. Item 1 measured perceived stress of the momentary situation on a dichotomous scale, adopted from a previous study of the relationship between stress reactivity and schizotypy dimensions (Chun et al., 2017). Each of the remaining 18 items was rated on a seven‐point Likert scale. Negative affect items were selected based on prior research examining stress reactivity in individuals at high risk for psychosis (Palmier‐Claus et al., 2012) and showed acceptable within‐ and between‐person reliability (ωʷ = 0.73; ω^b^  = 0.90). Mentalization items were adapted from the TAS to reflect momentary difficulties in identifying and describing feelings. Reliability of the three‐item mentalization scale was acceptable (ωʷ = 0.77; ω^b^  = 0.91). Psychotic experiences were assessed in the domains of paranoia, dissociation/intrusion and hallucinations. Paranoia items were sourced from Udachina et al. (2014), while the remaining psychotic experiences items were drawn from Myin‐Germeys et al. (2005). Past studies using the above psychotic experience items have shown acceptable internal consistency (Klippel et al., 2017; Myin‐Germeys et al., 2005), good convergent validity with interview‐based psychosis measures (Myin‐Germeys et al., 2005), and good concurrent validity with negative affect (Reininghaus et al., 2016). The 8‐item scale was consistent across time and between individuals (ωʷ = 0.69; ω^b^ = 0.91).

**TABLE 1:** The ESM items.

No.	Category	Items
1	Stress	How would you describe your current situation? (1 = positive, 2 = stressful)
2	Affect	I feel anxious
3	Affect	I feel restless
4	Affect	I feel irritated
5	Affect	I feel sad
6	Affect	I feel guilty
7	Affect	I feel embarrassed
8	Affect	I feel lonely
9	Mentalization	I am confused about what emotion I am feeling
10	Mentalization	I do not know what is going on inside me
11	Mentalization	I find it hard to express how I feel verbally
12	PE	I worry that others are plotting against me
13	PE	I feel that I can trust no‐one
14	PE	I believe that some people want to hurt me deliberately
15	PE	I hear things that aren't really there
16	PE	I see things that aren't really there
17	PE	I feel unreal
18	PE	I can't get these thoughts out of my head
19	PE	I feel like I am losing control

Abbreviation: PE, Psychotic experiences.

### Data analysis

All analyses were performed in RStudio 2023.06.1 (Posit team, 2023), and the R code used for statistical analysis is provided in the Supplementary Materials Appendix S1. The ESM data were structured as two‐level models, where repeated measurements (level 1) were nested within individuals (level 2). A compliance threshold was established at 30% of the repeated assessments, excluding participants who completed fewer than 17 assessments (Kirtley et al., 2021).

The ‘Lmer’ function from the *lme4* package (Bates et al., 2014) was used to estimate linear mixed effects and vector autoregressive models (VAR). Two mixed effects models were fitted. The initial model replicated the affective pathway hypothesis (Myin‐Germeys et al., 2003), examining whether momentary negative affective reactivity to stress and overall negative affect levels predicted concurrent psychotic experiences, with gender as an interaction term. To separate the between‐person and within‐person effects of negative affect on psychotic experiences, within‐person means of repeated negative affect measures were included. Momentary stress (a categorical variable) and gender (a three‐level factor) were set to interact with each other and with the concurrent predictor, negative affect. Autocorrelation of psychotic experiences was also controlled. The primary model built upon the initial affective pathway model by adding within‐person means and state‐level mentalizing difficulties as additional predictors, with random slopes specified for within‐person negative affect and mentalizing difficulties.

The mixed effect models assumed that standardized residuals followed a normal distribution and that no highly influential cases unduly impacted the results. Residual normality was assessed by examining a histogram and normal probability plot of residuals, with an expectation that 95% of standardized residuals fall within ±1.96 bounds. Cook's distance was calculated by dividing the sample size by 4 to determine a threshold for influential cases, with values exceeding this threshold indicating potential influence (Nieuwenhuis, 2012). A sensitivity analysis was conducted, in which the primary model was re‐estimated without the influential cases.

Multilevel VAR models were estimated to examine cross‐lagged associations among mentalizing difficulties, negative affect and psychotic experiences across time. In total, three models were tested, each structured to predict a single outcome variable based on its prior values and the other variables measured at the previous time point. For instance, momentary psychotic experiences were predicted based on prior values of psychotic experiences, mentalizing difficulties and negative affect. In the second and third models, mentalizing difficulties and negative affect were set as the respective outcome variables. Each predictor was entered using both raw and lagged values, with raw data used for outcome variables. Given the repeated testing across models, a Bonferroni correction was applied adjusting the significance threshold to *p* = .017 (0.05/3) to control for multiple comparisons.

## RESULTS

### Sample characteristics

Among 67 participants who enrolled in the study, four participants completed only the baseline assessment, and 20 participants were excluded due to response rates lower than 30%. The number of completed ESM responses for the analysed sample ranged from 17 to 52 (M = 25.95, SD = 11.84), with an average completion rate of 71%, and 33% across the full sample. The final sample (*n* = 43) was 63% female, 21% male and 16% non‐binary and aged between 18 and 38 (M = 28.8, SD = 4.7). Fifty‐three per cent of the sample were employed, 33% were students, and 14% unemployed. A personal history of psychosis was self‐reported by 30% of the sample, 24% reported a diagnosis of a psychotic disorder within the immediate family, and 37% reported receiving mental health support.

Differences between the included and excluded participants and between the participants with and without history of psychosis were examined for baseline assessments of disorganized schizotypy, negative symptoms, paranoia and indices of mentalizing difficulties. The only statistically significant difference was observed for the paranoia measure, whereby participants with a history of psychosis had higher scores compared to the participants without a history of psychosis (see Tables S1 and S2). This suggests that unusual experiences examined in this study are well represented in the sample.

Power calculations were performed using an online multilevel ESM power calculator (https://ekleiman.shinyapps.io/powercurves/), which estimates power based on observed study characteristics and an intraclass correlation coefficient (ICC) (Kleiman, 2021). Based on our final sample of 43 participants, 7‐day duration and 8 signals per day (maximum 56 responses per participant), and an adjusted ICC of 0.86 derived from our primary concurrent mixed effects model, we evaluated the detectable within‐person effect sizes. The power analysis indicated that the study design would achieve approximately 70% power to detect large within‐person effects (Cohen's *d* = 0.8) with a 71% completion rate observed in the study sample. However, power to detect medium effects (*d* = 0.5) remained below 60%, even under ideal completion conditions. This suggests that while the study was adequately powered to detect large within‐person associations, caution is warranted when interpreting null results for smaller effect sizes due to limited statistical power.

### Concurrent associations

The within‐ and between‐person correlations and descriptive statistics of the study variables are presented in Table 2. The mixed effects model testing the affective pathway hypothesis showed that, after accounting for autocorrelation, between‐person negative affect significantly predicted psychotic experiences (*b* = 0.42, SE = 0.10, *p* < .001), and momentary negative affect was a marginally significant predictor (*b* = 0.17, SE = 0.09, *p* = .058). Stress reactive increases in negative affect were associated with elevated psychotic experiences in female (*b* = 0.32, SE = 0.10, *p* < .001) and non‐binary participants (*b* = 0.41, SE = 0.13, *p* < .01). These effects became non‐significant after the inclusion of within‐ and between‐person mentalizing difficulties in the primary model. Accordingly, mentalizing difficulties at both state and trait levels were positively associated with psychotic experiences, and female and non‐binary participants reported significantly more psychotic experiences due to stress‐reactive decline in mentalization capacity than male participants. The results of the primary analysis are presented in Table 3.

**TABLE 2:** Within‐ and between‐person correlations and descriptive statistics of the study variables.

	Mentalizing	Negative affect	Psychotic experiences
Mentalizing	–	0.72	0.61
Negative affect	0.38	–	0.58
Psychotic experiences	0.34	0.48	–
Mean (SD)	3.11 (1.48)	3.19 (1.14)	2.12 (1.28)
Range	1–6.9	1.46–7	1–5.15

**TABLE 3:** Results of the primary mixed effects model of the concurrent associations between mentalizing, negative affect, and psychotic experiences as the outcome.

IV	*b*	SE	*T*	*p*
Intercept	0.64	0.39	1.62	.114
Psychotic experiences (lagged)	0.23	0.03	8.71	0.000
Negative affect (between‐person)	0.24	0.14	1.76	.087
Mentalizing (between‐person)	0.24	0.11	2.26	0.030
Negative affect (within‐person)	0.02	0.09	0.27	.788
Mentalizing (within‐person)	0.17	0.08	2.19	0.000
Situation: stressful	0.62	0.13	4.99	0.000
Momentary negative affect × stressful situation × female	0.11	0.10	1.09	.276
Momentary negative affect × stressful situation × non‐binary	−0.02	0.14	−0.13	.893
Momentary mentalizing × stressful situation × female	0.24	0.11	2.18	<.05
Momentary mentalizing × stressful situation × non‐binary	0.51	0.15	3.53	<.001

*Note*: ICC (unadjusted) = 0.35, ICC (adjusted) = 0.86. ICC reflects the proportion of between‐person variance in psychotic experiences.

Standardized residuals were all within the ±1.96 range, indicating that fewer than 5% fell outside the bounds expected under normality, consistent with the assumption of normally distributed residuals (see Figure S1A). While the standardized residuals all fell within ±1.96, indicating the absence of extreme outliers, the histogram (Figure S1B) suggested a leptokurtic distribution, and the normal probability plot (Figure S1C) revealed deviations from normality. These findings suggest that, although residuals were not unduly influential, the assumption of normally distributed residuals may have been violated. Additionally, Cook's distance cut‐off (4/43 = 0.09) was exceeded by three influential cases. These participants reported either a personal or family history of psychosis, so were retained for the reported analysis due to their relevance to the research context. Sensitivity analysis, conducted after removing the influential cases, revealed three significant associations, namely the autoregressive effect of psychotic experiences, between‐person effect of mentalizing difficulties, and an increase in psychotic experiences among non‐binary participants, driven by stress‐reactive elevations in mentalizing difficulties.

### Cross‐lagged associations

**FIGURE 2:**
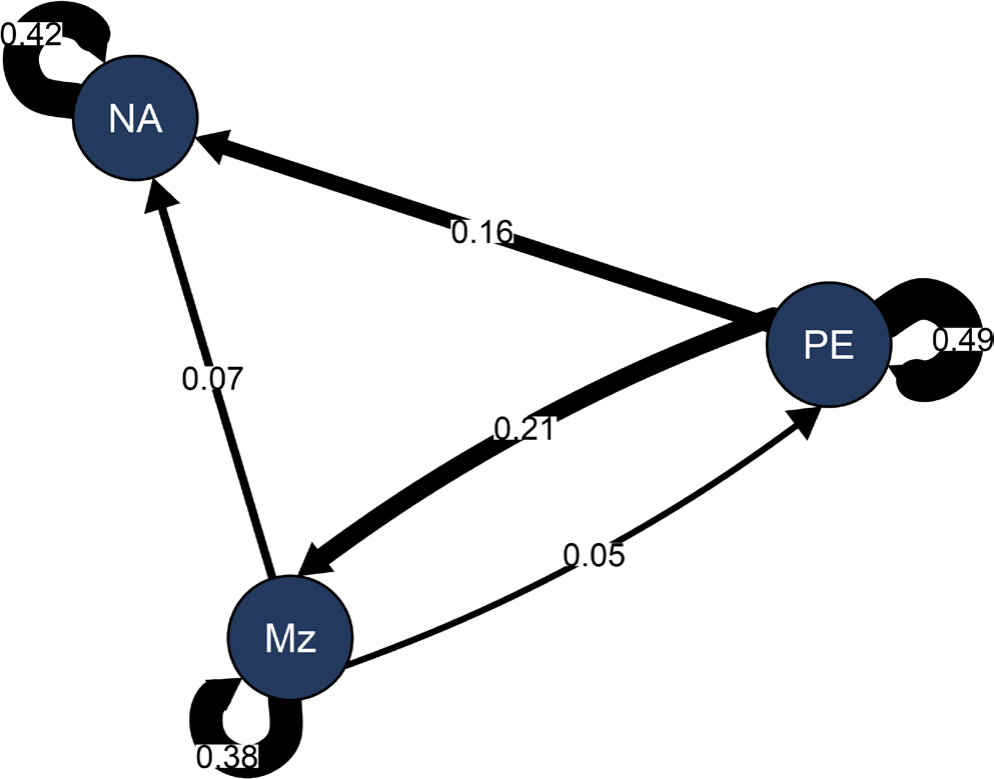
Network diagram of VAR model of mentalizing (Mz), negative affect (NA) and psychotic experiences (PE). Solid lines indicate significant directional relationships after Bonferroni adjustment *p* < .017, dashed line represents significance below *p* < .05, and curved arrows indicate autoregressive effects.

Figure 2 presents the network representation of the vector autoregressive model (VAR). The autoregressive effects revealed that psychotic experiences exhibited a relatively higher temporal stability over the week of measurement, suggesting that they were more consistent over time than mentalizing difficulties or negative affect. The cross‐lagged associations indicated that negative affect arose from the combined influence of prior psychotic experiences and mentalizing difficulties. However, the effect of mentalizing difficulties on subsequent psychotic experiences was very small, and its cross‐lagged association with negative affect became non‐significant following Bonferroni adjustment.

## DISCUSSION

The present study aimed to examine the moment‐to‐moment associations between mentalizing difficulties, negative affect and psychotic experiences as they unfold in daily life. We hypothesized that reflective disruptions under situational stress would predict the severity of concurrent psychotic experiences, with negative affect emerging as the outcome of the moment‐to‐moment feedback between psychotic experiences and inability to reflect on feelings. As hypothesized, mentalizing difficulties predicted the severity of concurrent and subsequent psychotic experiences, albeit with a very small positive effect size associated with subsequent psychotic experiences. Furthermore, our results indicate that, compared to male participants, female and non‐binary participants reported elevated psychotic experiences as a response to the concurrent difficulties in mentalizing due to stress. Our findings provide initial longitudinal evidence for theoretical assumptions regarding stress‐reactive decline in mentalizing capacity (Luyten et al., 2020) and the role of mentalizing difficulties in influencing the intensity of psychotic experiences (Debbané et al., 2016; Liotti & Gumley, 2008; Weijers et al., 2020).

Disruptions in mentalizing capacity have been characterized by stress reactive shifts from reflective processes towards biased and non‐reflective assumptions about self and others (Luyten et al., 2020). To our knowledge, stress‐reactivity of mentalizing capacity has been examined using ecological methods in only one previous study, in which stress predicted moment‐to‐moment mentalizing difficulties (Höller et al., 2025). Building on this, our study shows the clinical relevance of acute mentalizing difficulties in terms of its utility in predicting the severity of concurrent psychotic experiences. This also supports theoretical assumptions of mentalizing processes as moderators of psychosis expression (Debbané et al., 2016; Weijers et al., 2020), with florid psychosis emerging from a distress‐induced collapse of an already limited capacity for mentalization (Liotti & Gumley, 2008).

Understanding the nature of mentalizing difficulties, particularly distinguishing persistent inhibitions from stress‐reactive states, may help identify the processes of change attenuating psychosis risk. Alexithymia has been traditionally viewed as a profile of relatively stable trait impairments in identifying and describing feelings coupled with externally oriented thinking (Taylor & Bagby, 2013). Our findings suggest that the capacity to identify and describe feelings is stress‐reactive and thus better understood as state‐sensitive mentalizing processes. In contrast, the externally oriented thinking facet of alexithymia may reflect a more stable, trait‐like dimension, marked by a mentalizing capacity disengaged from inner experience and symbolic elaboration (Taylor et al., 2023). Meta‐analytic evidence supports the relevance of externally oriented thinking to psychological outcomes, showing moderate associations with schizophrenia diagnoses (Ozdemir, Xiao, et al., 2025). However, the clinical utility of externally oriented thinking remains constrained by the lack of valid and reliable measurement tools, which has contributed to inconsistent associations with other clinical outcomes (Hemming et al., 2019; Li et al., 2015; Norman et al., 2020). Despite this, externally oriented thinking retains conceptual value as a marker of enduring impairments in reflective functioning and warrants methodological refinement to fully capture its role in mentalizing‐related disorders.

Our findings also highlight the importance of attending to gender differences in state‐sensitivity of mentalizing difficulties. Current knowledge of gender differences in mentalizing difficulties (Caldarera et al., 2022) as well as psychosis (Barr et al., 2021; Carter et al., 2022; Riecher‐Rössler et al., 2018) remains limited. Our study suggested gender differences in the level of psychotic experiences emerged as a function of stress‐reactive mentalizing difficulties and that these effects were stronger for female and non‐binary participants. Similarly, women report more intense negative affective reactivity as a reaction to stress compared to men in the context of psychotic disorders (Myin‐Germeys et al., 2004). Conversely, men report higher proneness to externally oriented thinking compared to women (Peng et al., 2019; Vuillier et al., 2022). These patterns underscore the importance of disentangling psychosocial and biological determinants of gendered expressions of mentalizing and vulnerability to psychotic experiences.

Our findings suggest that fostering an adaptive mentalizing capacity may facilitate self‐regulation of unusual experiences. Yet the efficacy of promoting mentalization within psychosis populations requires further exploration, particularly within the matrix of phenomenological differences in self‐experiences across groups of psychosis vulnerability (Nelson et al., 2014). Psychotherapeutic efforts have been directed primarily at delusions and hallucinations with thought disorder (Wiesepape et al., 2023) and negative symptoms (Galderisi et al., 2021) remaining underexplored due to challenges in establishing a therapeutic alliance (Palmier‐Claus et al., 2017). Mentalizing capacity of the clinician emerges as an important determinant in regulating implicit or explicit reactions of clinicians to psychotic experiences. The clinician's approach towards unusual self‐experiences—whether with care or avoidance—plays a pivotal role in either fostering or hindering this alliance, determining therapeutic outcomes.

### Limitations and future directions

A number of methodological and design issues limit generalizability of the findings, including sampling method, sample size and measurement of mentalizing difficulties. Although data were collected from the general population, participants with and without a history of psychosis did not differ significantly in baseline levels of negative symptoms, disorganized schizotypy or mentalizing difficulties, suggesting that the sample may reflect characteristics of a psychosis risk sample. However, the study may have been underpowered to detect small to medium within‐person effects. These sampling issues, coupled with the sensitivity of findings to influential cases, underscore the need for replication in larger, clinically ascertained samples.

The self‐report items evaluating mentalizing difficulties in daily life are novel in the context of ecological momentary assessment and have not been validated. A criticism of self‐report measurements of mentalizing difficulties involves their large positive associations with negative affect taken to suggest that self‐reported mentalizing difficulties may reflect general psychological distress more than an inability to identify and describe emotional experiences associated with distress (Lane et al., 2015; Leising et al., 2009; Marchesi et al., 2014). In the current study, the momentary mentalization items had acceptable reliability estimates and showed distinct associations with psychotic experiences when negative affect was controlled for. The psychometric evidence therefore supports the construct validity of the study items used to assess momentary mentalizing difficulties in identifying and describing feelings. However, stepwise testing of the temporal model warrants further investigation with a joint multivariate VAR (i.e., Epskamp et al., 2018), potentially accounting for diurnal variations in sleep patterns as a covariate.

## CONCLUSION

This study is the first to capture the moment‐to‐moment relationship between mentalizing difficulties and psychotic experiences, offering important real‐world evidence in support of the view that mentalizing difficulties actively contribute to the ongoing maintenance of psychotic experiences. The results highlight the clinical utility of a state‐sensitive approach to mentalizing, particularly in identifying and describing feelings, for predicting psychotic experiences and informing timely process‐oriented interventions. Future research using longitudinal designs could disentangle the interplay between dispositional and reactive components of mentalizing across clinical and non‐clinical populations, while also attending to identity‐related factors such as gender and cultural diversity.

## AUTHOR CONTRIBUTIONS

**Ercan Ozdemir:** Conceptualization; methodology; data curation; investigation; visualization; writing – review and editing; writing – original draft; project administration; formal analysis. **Angus MacBeth:** Conceptualization; methodology; supervision; writing – review and editing. **Helen Griffiths:** Conceptualization; methodology; supervision; writing – review and editing.

## CONFLICT OF INTEREST STATEMENT

The authors declare no conflicts of interest

## Supporting information


Appendix S1

Appendix S2

## Data Availability

The data that support the findings of this study are available from the corresponding author upon reasonable request.
