# Supplementary material for: Mentalizing under stress and psychotic experiences: An experience sampling study
Source: Psychol Psychother. 2026 Feb 27;99(2):669–83. doi: 10.1111/papt.70048 (PMC13162178; doi:10.1111/papt.70048)
Supplement: Supplementary file 1 — Appendix S1 [file PAPT-99-669-s002.docx]

Mentalizing under Stress and Psychotic Experiences: An Experience Sampling Study Ozdemir et al. (2025)

library(influence.ME) # cook's distance
library(GPArotation)
library(data.table)
library(tidyverse)
library(ggplot2)
library(lme4)
library(lmerTest)
library(psych) #for PCA
library(tidyselect)
library(knitr)
library(performance) #for icc
library(readxl)
library(esmpack)
library(misty) #multilevel correlation
library(car)
library(simr)
library(Cairo)
library(qgraph)

#Load Data

dat<- read_xlsx("dat.xlsx")

# Count total number of valid responses per participant
participants <- dat %>%
 group_by(id) %>%
 summarise(total_responses = sum(!is.na(`Tapped in the notification bar`)))
# Mean and standard deviation of total responses
mean_responses <- mean(participants$total_responses, na.rm = TRUE)
sd_responses <- sd(participants$total_responses, na.rm = TRUE)

# Print results
cat("Mean total responses:", mean_responses, "\n")
cat("SD of total responses:", sd_responses, "\n")
# Calculate total actual responses across all participants
total_actual_responses <- sum(participants$total_responses)

# Define total possible responses
n_participants <- 43 # Total enrolled participants
prompts_per_day <- 8
n_days <- 7
total_possible_responses <- n_participants * prompts_per_day * n_days

# Compute overall completion rate
completion_rate <- total_actual_responses / total_possible_responses

# Print completion rate as percentage
completion_percentage <- completion_rate * 100
print(paste0("Overall completion rate: ", round(completion_percentage, 2), "%"))

#demographics

round(mean(get.timeinvar(Age, id, data=dat)), digits=1)
round(sd(get.timeinvar(Age, id, data=dat)), digits=1)
round(range(get.timeinvar(Age, id, data=dat)), digits=1)

table(get.timeinvar(Gender, id, data=dat)) #gender distribution
round(prop.table(table(get.timeinvar(Gender, id, data=dat))) * 100, digits=1)# as percentages

table(get.timeinvar(ExpOfPsychosis, id, data=dat)) # Distribution of self-reported psychosis history
round(prop.table(table(get.timeinvar(ExpOfPsychosis, id, data=dat))) * 100, digits=1)

table(get.timeinvar(FamilyHealth, id, data=dat)) #family history of psychosis

table(get.timeinvar(Treatment, id, data=dat)) #mental health support
round(prop.table(table(get.timeinvar(Treatment, id, data=dat))) * 100, digits=1)

# Variable formation

dat$situation <- as.factor(dat$situation) #1 = pleasant 2 = stressful
dat$Gender <- as.factor(dat$Gender) # 1= male - 2 = female / 4 = non-binary

#Mentalizing
dat$ment <- combitems(c("DIF.conf", "DIF.inside", "DDF"), data=dat)
dat$mmentpl <- calc.mean(ment, id, data=dat, expand=TRUE) # compute person-level means
dat$cmentpl <- calc.mcent(ment, id, data=dat) #compute within-person mean centered mentalizing
dat$rmentlag <- lagvar(ment, id=id, day=day, data=dat) # mentalizing lagged

#Psychotic experiences
dat$pe <- combitems(c("par1", "par2", "par3", "int.thoughts","unreal", "loss.control", "hal.hear", "hal.see"), data=dat)
dat$mpepl <- calc.mean(pe, id, data=dat, expand=TRUE)
dat$cpepl <- calc.mcent(pe, id, data=dat)
dat$rpelag <- lagvar(pe, id=id, day=day, data=dat)

#negative affect
dat$na <- combitems(c("anxious", "irritated", "sad", "guilty", "embarrassed", "restless", "lonely"), data=dat)
dat$mnapl <- calc.mean(na, id, data=dat, expand=TRUE)
dat$cnapl <- calc.mcent(na, id, data=dat)
dat$rnalag <- lagvar(na, id=id, day=day, data=dat)

#Psychometrics

##Descriptives

#mentalization
mean(get.timeinvar(mmentpl, id, data=dat))
sd(get.timeinvar(mmentpl, id, data=dat))
round(range(get.timeinvar(mmentpl, id, data=dat)), digits=1)

#negative affect
mean(get.timeinvar(mnapl, id, data=dat))
sd(get.timeinvar(mnapl, id, data=dat))
range(get.timeinvar(mnapl, id, data=dat))

#Psychotic experiences
mean(get.timeinvar(mpepl, id, data=dat))
sd(get.timeinvar(mpepl, id, data=dat))
range(get.timeinvar(mpepl, id, data=dat))

## multilevel correlations

multilevel.cor(dat[, c("ment", "na", "pe" )],
 cluster = dat$id)

## multilevel reliability

multilevel.omega(dat[,c("DIF.conf", "DIF.inside", "DDF")], cluster = dat$id,missing = "listwise", write = "Mentalizing Multilevel_Omega.xlsx")

multilevel.omega(dat[,c("par1", "par2", "par3", "int.thoughts","unreal", "loss.control", "hal.hear", "hal.see")], cluster = dat$id,missing = "listwise", write = "Psychosis Multilevel_Omega.xlsx")

multilevel.omega(dat[,c("anxious", "irritated", "sad", "guilty", "embarrassed", "restless", "lonely")], cluster = dat$id,missing = "listwise", write = "Affect Multilevel_Omega.xlsx")

#Concurrent assocations

## replication of the affective pathway model

affpathmod <- lmer(pe ~ rpelag + mnapl + cnapl*situation*Gender + (1 + cnapl |id), dat, control = lmerControl(optimizer = "bobyqa"))
summary(affpathmod)
icc(affpathmod)

## Hypothesised model

mod <- lmer(pe ~ rpelag + mnapl + mmentpl + (cnapl + cmentpl)*situation*Gender + (1 + cnapl+ cmentpl |id), dat, control = lmerControl(optimizer = "bobyqa"))
summary(mod)
icc(mod)

## assessment of model assumptions

sav <- influence.ME::influence(mod, "id")

cds <- cooks.distance(sav)
plot(cds, type="o", pch=19)
abline(h=0.02, lty="dotted")
names(cds) <- sav$deleted #attach subject labels to values
cds[which(cds > 0.09)] #which subjects have a Cook's distance above 0.09? (4/43 participants= .09)
infl <- subset(cds, cds > 0.09) #Outliers
infl

#PLOTS

fitval <- fitted(mod)
stdres <- resid(mod, type="pearson")

jpeg("fitted vs stand residuals.jpeg", width=10, height=8, res=1200, units="in")
plot(fitval, stdres, pch=19, cex=0.5, ylim=c(-3.5,3.5))
abline(h=0)
abline(h=c(-1.96,1.96), lty="solid")
dev.off()

## plot fitted values versus standardized residuals with jitter
jpeg("fitted vs stand residuals jittered.jpeg", width=10, height=8, res=1200, units="in")
plot(jitter(fitval, amount=0.2), jitter(stdres, amount=0.3), pch=19, cex=0.5, ylim=c(-3.5,3.5))
abline(h=0)
abline(h=c(-1.96,1.96), lty="dotted")

dev.off()
## what % of the standardized residuals is > than 1.96 in absolute value?
round(100 * mean(abs(stdres) > 1.96), digits=5) #0%

## histogram of the standardized residuals
hist(stdres, main="", xlab="Standardized Residual")

## histogram of the standardized residuals (with normal distribution superimposed)
jpeg("histogram std res.jpeg", width=10, height=8, res=1200, units="in")
hist(stdres, breaks=50, main="", xlab="Standardized Residual", freq=FALSE)
curve(dnorm(x, mean=mean(stdres), sd=sd(stdres)), add=TRUE, lwd=2)
dev.off()

## normal probability plot of the standardized residuals
jpeg("normal prob plot.jpeg", width=10, height=8, res=1200, units="in")
qqnorm(stdres, pch=19, cex=0.5)
qqline(stdres)
dev.off()

#Vector autoregressive model of the cross-lagged relationships

# model 1 predicting PE
peCL <- lmer(pe ~ rnalag + rmentlag + rpelag + (1 | id), dat, REML = F, control = lmerControl(optimizer = "bobyqa"))
summary(peCL)
icc(peCL)


# model 2 predicting Mentalizing
mentCL <- lmer(ment ~ rnalag + rmentlag + rpelag + (1 |id), dat, REML = F, control = lmerControl(optimizer = "bobyqa"))
summary(mentCL)
icc(mentCL)


# model 3 predicting NA
naCL <- lmer(na ~ rnalag + rmentlag + rpelag + (1 |id), dat, REML = F, control = lmerControl(optimizer = "bobyqa"))
summary(naCL)
icc(naCL)

#covariance
cov(residuals(peCL),residuals(mentCL))
cov(residuals(peCL),residuals(naCL))
cov(residuals(naCL),residuals(mentCL))

## network graph

# collect slopes into a matrix
sav <- cbind(fixef(naCL), fixef(mentCL), fixef(peCL))[-1,]
colnames(sav) <- c("NA", "Mz", "PE")
round(sav, digits=2)
sav <- sav[-c(4:7),]
sav

# collect all p-values into a matrix and apply Bonferroni correction
pvals <- cbind(coef(summary(naCL))[,"Pr(>|t|)"], coef(summary(mentCL))[,"Pr(>|t|)"], coef(summary(peCL))[,"Pr(>|t|)"] )[-1,]
colnames(pvals) <- colnames(sav)
sav[pvals > 0.027] <- 0
sav <- sav[-c(4:7),]

# Define the line types for each edge, with '2' for dotted
lty_matrix <- matrix(1,
 nrow = nrow(sav),
 ncol = ncol(sav),
 dimnames = list(rownames(sav), colnames(sav)))
lty_matrix[2, 1] <- 2 # Set the specific edge as dotted

# create network graph

CairoTIFF("network plot.tiff", width = 4000, height = 3000, res = 300)
qgraph(sav, mar=rep(12,4),lty = lty_matrix, labels=colnames(sav), edge.labels=TRUE, curve=2, color = "#23395d",theme = "gimme", edge.color = "black", edge.width = 2)
dev.off()

## conceptual model

# Define variable names
vars <- c("PE", "Mz", "NA")

# Create a 3x3 matrix of zeros
coef_mat <- matrix(0, nrow = 3, ncol = 3)
colnames(coef_mat) <- rownames(coef_mat) <- vars

# Define directed paths
coef_mat["PE", "Mz"] <- 0.1 # PE → Mz
coef_mat["Mz", "PE"] <- 0.1 # Mz → PE
coef_mat["Mz", "NA"] <- 0.2 # Mz → NA
coef_mat["PE", "NA"] <- 0.2 # PE → NA

# Manual layout (triangular)
layout_matrix <- matrix(c(
 -1, -1, # PE at bottom left
 0, 1, # Mz at top center
 1, -1 # NA at bottom right
), nrow = 3, ncol = 2, byrow = TRUE)

# Plot
CairoTIFF("conceptual_model.tiff", width = 4000, height = 3000, res = 300)
qgraph(coef_mat,
 layout = layout_matrix,
 directed = TRUE,
 labels = vars,
 theme = "gimme",
 color = "#23395d",
 edge.color = "black",
 edge.width = .5,
 curve = 2,
 mar = rep(12,4))
dev.off()
