# Supplementary material for: Mentalizing under stress and psychotic experiences: An experience sampling study
Source: Psychol Psychother. 2026 Feb 27;99(2):669–83. doi: 10.1111/papt.70048 (PMC13162178; doi:10.1111/papt.70048)
Supplement: Supplementary file 2 — Appendix S2 [file PAPT-99-669-s001.docx]

**Supplementary Materials**

**Mentalizing under Stress and Psychotic Experiences: An Experience Sampling Study**

**Supplementary Tables**

| **Table 7.2** Independent samples t-test results for the difference between included **Table S1.** Independent samples t-test results for the difference between included and excluded participants | | | | |
| --- | --- | --- | --- | --- |
| Variables | **Included**  (n = 43)  Mean (SD) | **Excluded**  (n = 24)  Mean (SD) | t (df) | P |
| TAS-20 | 53.83 (12.20) | 54.07 (14.83) | -0.07 (63) | 0.946 |
| Disorganized schizotypy | 4.26 (4.16) | 4.70 (4.26) | 0.40 (63) | 0.691 |
| Negative symptoms | 37.22 (9.56) | 36.17 (10.31) | -0.41 (63) | 0.684 |
| Mental state uncertainty | 1.37 (0.54) | 1.62 (0.69) | 1.63 (63) | 0.107 |
| Paranoia | 32.33 (15.64) | 34.73 (16.65) | 0.57 (62) | 0.571 |

| **Table S2.** Independent samples t-test results for the difference between participants with and without history of psychosis | | | | |
| --- | --- | --- | --- | --- |
| Variables | **Psychosis** Mean (SD) | **Non-psychosis**  Mean (SD) | t (df) | P |
| TAS-20 | 57.16 (15.57) | 52.34 (14.61) | 0.94 (39) | 0.351 |
| Disorganized schizotypy | 5.67 (4.40) | 3.45 (3.81) | 1.62 (39) | 0.493 |
| Negative symptoms | 40.08 (9.25) | 35.89 (9.73) | 1.27 (38) | 0.213 |
| Mental state uncertainty | 1.24 (0.72) | 0.87 (0.61) | 1.65 (39) | 0.108 |
| Paranoia | 39.58 (18.77) | 25.14 (9.42) | 2.30 (39) | **< 0.01** |

**Supplementary Figures**

**(B)**

**(B)**

**(B)**

**(B)**

**(B)**

**(B)**

**(B)**

**(B)**

**(B)**

**(B)**

**(B)**

**(B)**

**(B)**

**(B)**

**(B)**

**(B)**

**(B)**

**(B)**

**(B)**

**(B)**

**(B)**

**(B)**

**(B)**

**(B)**

**(B)**

**(B)**

**(B)**

**(B)**

**(B)**

**(B)**

**(B)**

**(B)**

**(B)**

**(B)**

**(B)**

**(B)**

**(B)**

**(B)**

**(B)**

**(B)**

**(B)**

**(B)**

**(B)**

**(B)**

**(B)**

**(B)**

**(B)**

**(B)**

**(B)**

**(B)**

**(B)**

**(B)**

**(B)**

**(B)**

**(B)**

**(B)**

**(B)**

**(B)**

**(B)**

**(B)**

**(B)**

**(B)**

**(B)**

**(B)**

**(B)**

**(B)**

**(B)**

**(B)**

**(B)**

**(B)**

**(B)**

**(B)**

**(B)**

**(B)**

**(B)**

**(B)**

**(B)**

**(B)**

**(B)**

**(B)**

**(B)**

**(B)**

**(B)**

**(B)**

**(B)**

**(B)**

**(B)**

**(B)**

**(B)**

**(B)**

**(B)**

**(B)**

**(B)**

**(B)**

**(B)**

**(B)**

**(B)**

**(B)**

**(B)**

**(B)**

**(B)**

**(B)**

**(B)**

**(B)**

**(B)**

**(B)**

**(B)**

**(B)**

**(B)**

**(B)**

**(B)**

**(B)**

**(B)**

**(B)**

**(B)**

**(B)**

**(B)**

**(B)**

**(B)**

**(B)**

**(B)**

**(B)**

**(B)**

**(B)**

**(B)**

**(B)**

**(B)**

**(B)**

**Figure S1.** Plots for the evaluation of model assumptions

(A) the plot of fitted values versus standardized residuals, (B) histogram of the standardized residuals with normal distribution superimposed, (C) normal probability plot of the standardized residuals

**(A)**

**(A)**

**(A)**

**(A)**

**(A)**

**(A)**

**(A)**

**(A)**

**(A)**

**(A)**

**(A)**

**(A)**

**(A)**

**(A)**

**(A)**

**(A)**

**(A)**

**(A)**

**(A)**

**(A)**

**(A)**

**(A)**

**(A)**

**(A)**

**(A)**

**(A)**

**(A)**

**(A)**

**(A)**

**(A)**

**(A)**

**(A)**

**(A)**

**(A)**

**(A)**

**(A)**

**(A)**

**(A)**

**(A)**

**(A)**

**(A)**

**(A)**

**(A)**

**(A)**

**(A)**

**(A)**

**(A)**

**(A)**

**(A)**

**(A)**

**(A)**

**(A)**

**(A)**

**(A)**

**(A)**

**(A)**

**(A)**

**(A)**

**(A)**

**(A)**

**(A)**

**(A)**

**(A)**

**(A)**

**(A)**

**(A)**

**(A)**

**(A)**

**(A)**

**(A)**

**(A)**

**(A)**

**(A)**

**(A)**

**(A)**

**(A)**

**(A)**

**(A)**

**(A)**

**(A)**

**(A)**

**(A)**

**(A)**

**(A)**

**(A)**

**(A)**

**(A)**

**(A)**

**(A)**

**(A)**

**(A)**

**(A)**

**(A)**

**(A)**

**(A)**

**(A)**

**(A)**

**(A)**

**(A)**

**(A)**

**(A)**

**(A)**

**(A)**

**(A)**

**(A)**

**(A)**

**(A)**

**(A)**

**(A)**

**(A)**

**(A)**

**(A)**

**(A)**

**(A)**

**(A)**

**(A)**

**(A)**

**(A)**

**(A)**

**(A)**

**(A)**

**(A)**

**(A)**

**(A)**

**(A)**

**(A)**

**(A)**

**(A)**


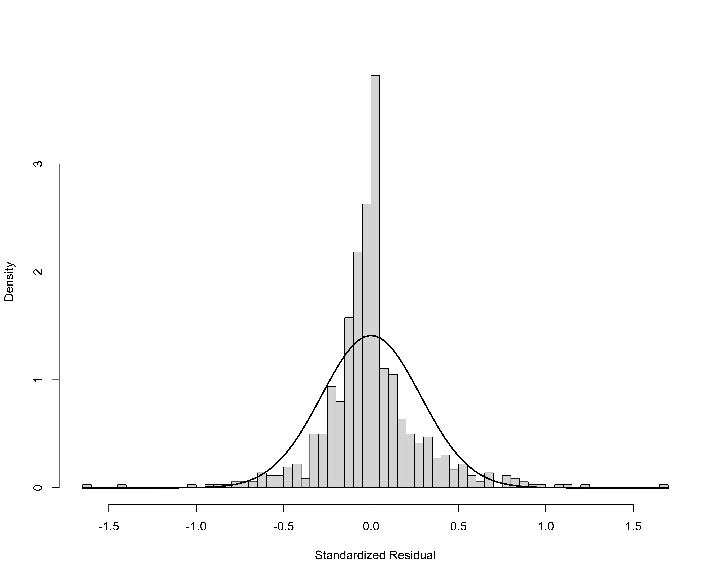

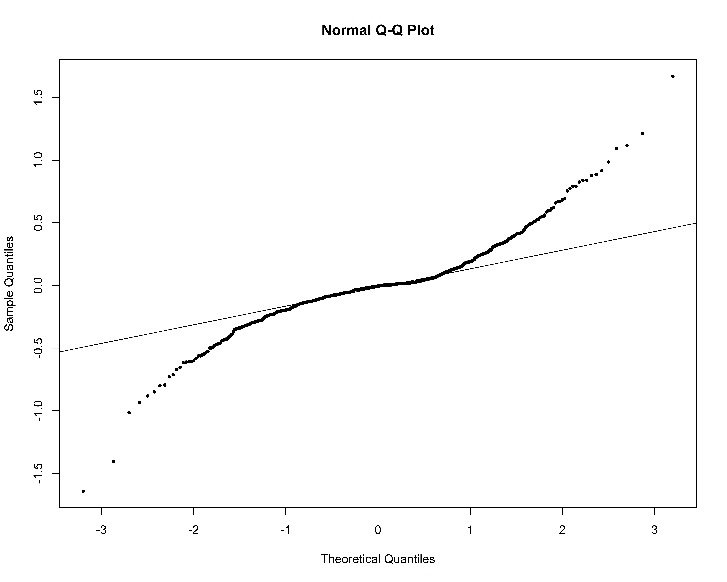


**(C)**

**(C)**

**(C)**

**(C)**

**(C)**

**(C)**

**(C)**

**(C)**

**(C)**

**(C)**

**(C)**

**(C)**

**(C)**

**(C)**

**(C)**

**(C)**

**(C)**

**(C)**

**(C)**

**(C)**

**(C)**

**(C)**

**(C)**

**(C)**

**(C)**

**(C)**

**(C)**

**(C)**

**(C)**

**(C)**

**(C)**

**(C)**

**(C)**

**(C)**

**(C)**

**(C)**

**(C)**

**(C)**

**(C)**

**(C)**

**(C)**

**(C)**

**(C)**

**(C)**

**(C)**

**(C)**

**(C)**

**(C)**

**(C)**

**(C)**

**(C)**

**(C)**

**(C)**

**(C)**

**(C)**

**(C)**

**(C)**

**(C)**

**(C)**

**(C)**

**(C)**

**(C)**

**(C)**

**(C)**

**(C)**

**(C)**

**(C)**

**(C)**

**(C)**

**(C)**

**(C)**

**(C)**

**(C)**

**(C)**

**(C)**

**(C)**

**(C)**

**(C)**

**(C)**

**(C)**

**(C)**

**(C)**

**(C)**

**(C)**

**(C)**

**(C)**

**(C)**

**(C)**

**(C)**

**(C)**

**(C)**

**(C)**

**(C)**

**(C)**

**(C)**

**(C)**

**(C)**

**(C)**

**(C)**

**(C)**

**(C)**

**(C)**

**(C)**

**(C)**

**(C)**

**(C)**

**(C)**

**(C)**

**(C)**

**(C)**

**(C)**

**(C)**

**(C)**

**(C)**

**(C)**

**(C)**

**(C)**

**(C)**

**(C)**

**(C)**

**(C)**

**(C)**

**(C)**

**(C)**

**(C)**

**(C)**

**(C)**

**(C)**

**
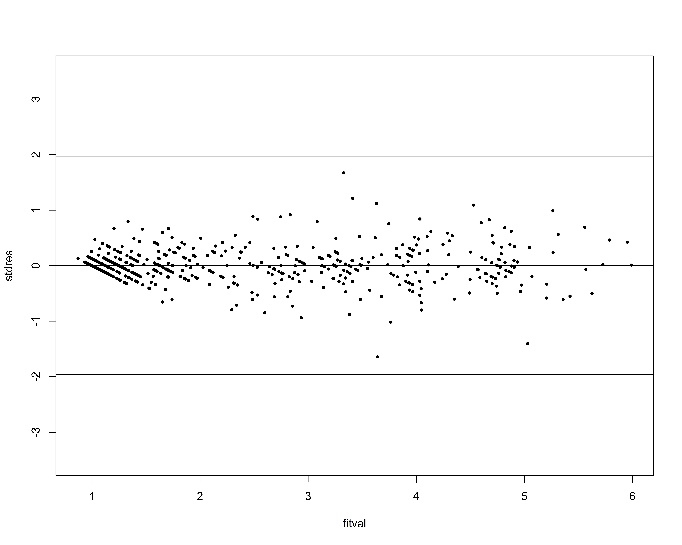
**
